# Messenger RNA levels of podocyte-associated proteins in subjects with different degrees of glucose tolerance with or without nephropathy

**DOI:** 10.1186/1471-2369-14-214

**Published:** 2013-10-08

**Authors:** Jonathan Fraportti do Nascimento, Luis H Canani, Fernando Gerchman, Patricia G Rodrigues, Gabriel Joelsons, Mariane dos Santos, Sane Pereira, Francisco V Veronese

**Affiliations:** 1 Post Graduate Program in Medicine: Medical Sciences, Universidade Federal do Rio Grande do Sul, Porto Alegre, RS, Brazil; 2 Division of Endocrinology, Hospital de Clínicas de Porto Alegre, Porto Alegre, RS, Brazil; 3 Division of Nephrology, Hospital de Clínicas de Porto Alegre, Ramiro Barcelos 2350, Porto Alegre, RS ZIP 90035-003, Brazil

**Keywords:** Diabetic nephropathy, Prediabetes, Podocyte, Nephrin, TRPC6

## Abstract

**Background:**

To investigate gene expression of podocyte-specific proteins in urine of diabetes and prediabetes subjects and the association of these proteins with albuminuria.

**Methods:**

Fifteen controls, 19 prediabetes, and 67 diabetes subjects were included. Messenger RNA of nephrin, podocin, podocalyxin, synaptopodin, TRPC6, alpha-actinin-4, and TGF-β_1_ were measured using RT-PCR. Podocyte marker expression was correlated with albuminuria, glycemic control, and renal function. The diagnostic performance of the genes used to detect increased albuminuria was assessed using ROC curves and Poisson regressions.

**Results:**

Podocyte marker expression was significantly higher in diabetic subjects. Urinary nephrin was correlated with increasing levels of albuminuria; risk of albuminuria increased by 20% for every one-unit increase in the log10 of nephrin mRNA. Nephrinuria was found in 53%, 71%, and 90% of normo-, micro-, and macroalbuminuric diabetes subjects, respectively (p = 0.023). Urinary nephrin, podocalyxin, TRPC6, podocin, and alpha actinin-4 were correlated with glycemic control and albuminuria but not with renal function.

**Conclusions:**

Diabetes subjects had higher urinary mRNA levels of podocyte proteins than nondiabetic subjects, even the normoalbuminuric patients. Nephrinuria was correlated with diabetic nephrophathy stage and predicted pathological albuminuria. Urinary mRNA levels of podocyte markers of prediabetic subjects did not differ from controls.

## Background

Diabetic nephropathy (DN) is the leading cause of end-stage renal disease (ESRD) worldwide. In Brazil, DN is the etiology of ESRD in 28% of hemodialysis patients [1]. The pathogenesis of DN is not yet fully understood, and recent studies have focused on the mechanisms of glomerular podocyte injury [2,3].

Microalbuminuria (MI) is a known early marker of diabetes mellitus (DM)-induced damage to the glomerular filtration barrier. Clinical and experimental studies have suggested that the onset of albuminuria is associated with podocyte injury involving podocyte hypertrophy and effacement [3,4]. The mechanisms of injury in DN include glomerular hyperfiltration, toxicity of active oxygen specimens and advanced glycation end-products, angiotensin II action, and cytokine and growth factor secretion [5-7]. Podocytes detach from the glomerular basement membrane (GBM) and are shed into the urine as viable and/or apoptotic cells that can be detected by different laboratory techniques [8,9].

Analyzing the gene expression of podocyte proteins has increased our understanding of the pathogenesis of proteinuria in DN. Slit diaphragm nephrin and podocin are closely linked to cytoskeletal alpha actinin-4 and synaptopodin, enabling dynamic rearrangements of the podocyte architecture. Podocalyxin at the apical membrane, a phenotypic marker of the podocyte cell, limits the passage of negatively charged albumin [10]. Transient receptor potential calcium channel 6 (TRPC6) is another protein located at the luminal membrane that regulates intracellular calcium concentration; its induction disrupts the actin cytoskeleton, resulting in the proteinuria of acquired kidney disease [11]. In more advanced stages of DN, TGF-β_1_, which induces podocyte apoptosis, has been correlated with fibrosis and chronic intra-renal damage [12].

The expression of podocyte markers in glomerular disease has been investigated using immunostaining or molecular analysis to quantitatively measure protein and mRNA levels respectively in renal tissue. However, renal biopsy is usually not indicated for the diagnosis of DN. Recently, the detection of increased urinary mRNA of podocyte-specific molecules in DN and other glomerular diseases has emerged as a useful, non-invasive tool to assess podocyturia as a signal of podocyte damage and disease activity [9,13,14]. For example, urinary mRNA of nephrin and podocin measured by real time polymerase chain reaction (RT-PCR) [15] or fragments of nephrin in urine analyzed by Western Bloting [16] correlated with the albumin excretion and the glomerular filtration rate in DN. In addition, oral hypoglycemic agents and ARB-2 have been clinically and experimentally shown to reduce podocyte injury and podocyturia [14,17,18].

Whether podocyte injury and podocyturia occur at the very early stages of glucose intolerance, namely during the prediabetic stage, is a topic of speculation; however, this hypothesis has not yet been tested in clinical studies. In this study, we compared the mRNA levels of podocyte-specific proteins of subjects with diabetes, prediabetes and in healthy individuals to verify whether the excretion of podocytes in urine varies throughout the different stages of DN and is already increased in early stages of glucose intolerance.

## Methods

### Study population

From December 2010 to January 2012, we recruited subjects with different degrees of glucose tolerance, with and without increased urinary albumin excretion rates (UAER).

### Study procedures and assay

Demographic, clinical, and laboratory data were collected from medical records. A standard 75 g oral glucose tolerance test (OGTT) was performed according to the American Diabetes Association protocol. Prediabetes was diagnosed according to the OGTT plasma glucose (PG) as either impaired fasting glucose (fasting PG 110–125 mg/dL and 2-hr PG <140 mg/dL) or impaired glucose tolerance (fasting PG <100 mg/dL and 2-hr PG 140–199 mg/dL). Diabetes was diagnosed by fasting PG ≥126 mg/dL and/or 2-hr PG ≥ 200 mg/dL [19].

Subjects with DM type 1 or type 2 were divided into three groups according to their urinary excretion of albumin: normoalbuminuria (NO) (<30 mg/g creatinine; n = 34); MI (30–300 mg/g creatinine; n = 14); or macroalbuminuria (MA) (>300 mg/g creatinine; n = 19).

Patients with unstable renal function, defined by a reduction of the estimated glomerular filtration rate in the previous six months, acute cardiovascular events, urinary tract infections, or any acute medical condition were excluded from the study.

The glomerular filtration rate (eGFR) was estimated using the Chronic Kidney Disease Epidemiology Collaboration (CKD-EPI) equation [20], and determined as the mean of three measurements. Urinary albumin excretion was measured in a random sample using an immunoturbidimetric method. Glycated hemoglobin (HbA1c) was measured using high-performance liquid chromatography and reported in NGSP units (%) and IFCC (International Federation of Clinical Chemistry) units (mmol/mol). Glycated hemoglobin was used as a measure of glycemic control.

The sample size was calculated using the WINPEPI version 9.7 [21]. Based on a pilot study from our laboratory, we defined healthy individuals and subjects with diabetes respectively by a mean ± SD value for urinary nephrin log10 mRNA of 1.9 ± 0.8 and 3.5 ± 1.4. To achieve a 90% study power and a 5% level of significance, a sample of at least 67 subjects with diabetes and 15 controls was required.

The study was approved by the institutional review board at Hospital de Clínicas de Porto Alegre (HCPA). All subjects agreed to participate in the study and signed an informed consent form.

### Quantification of mRNA levels in the urine sediment

The mRNA level of podocyte-associated proteins was quantitatively measured in the urinary sediment cells in early morning urine specimens (whole stream). The gene expression of nephrin, podocin, alpha-actinin-4, synaptopodin, podocalyxin, and TRPC6 was measured. As these biomarkers are specifically expressed on podocyte cells and can be assessed by RT-PCR in the urinary sediment [13], podocyturia was defined by an increased urinary mRNA expression in relation to the levels found in healthy individuals, as described in other studies [15]. TFG-β_1_, a marker of kidney fibrosis that actively participates in the pathophysiology of DN, was included in the molecular analysis. The expression of urinary podocyte biomarkers was compared between type 1 and type 2 diabetes subjects.

### mRNA extraction and complementary DNA transcription

Material was extracted from urine samples using the QIAamp® RNA Blood Mini Kit (Qiagen Inc. Chatsworth, CA, USA) according to the manufacturer’s instructions. Urine samples were centrifuged at 1,800 rpm for 10 minutes. The supernatant was discarded, and the pellet was resuspended with buffered saline and centrifuged at 10,000 rpm for 10 minutes before being stored at -80°C until use. Total RNA was quantified with a NanoDrop® 1000 Spectrophotometer v. 3.7 (Thermo Fisher Scientific, Wilmington, DE, USA). The ratio of absorbance at 260/280 nm was used to assess RNA purity. Reverse transcription of total RNA was performed using the High-Capacity cDNA Kit (Applied Biosystems, Foster City, CA, USA) according to the manufacturer’s instructions. The final volume of purified RNA was 20 μL, and it was stored at -20°C.

### Real-time polymerase chain reaction

RT-PCR was performed using the Taqman® Universal PCR Master Mix. This mix contains AmpliTaq Gold® DNA Polymerase, AmpErase® UNG, ROX passive reference, buffer, and dNTPs (Applied Bioystems, Foster City, CA, USA), as well as gene-specific primers for the mRNA amplification of the following genes (all from Applied Bioystems, Foster City, CA, USA): NPSH1, nephrin (ID: Hs00190446_m1); NPSH2, podocin (ID: Hs00387817_m1); podocalyxin (ID: Hs01574644_m1); synaptopodin (ID: Hs00326493_m1); alpha-actinin-4 (ID: Hs00245168_m1); TRPC6 (ID: Hs00395102_m1); and TGF-β_1_ (ID: Hs00998133_m1). In addition, 18 s rRNA (Taqman® PDAR, Foster City, CA, USA) was used as an endogenous control to correct for variations in the samples. RT-PCR was performed in duplicate in 96-well plates containing 2 μL of cDNA. The thermal conditions of the cycles were 50°C for 2 minutes, 60°C for 30 minutes, and 95°C for 5 minutes, which was followed by 40 cycles at 94°C for 20 seconds and 62°C for 60 seconds. The data were collected in the ABI PRISM SDS 7000 thermal cycler (Applied Bioystems, Foster City, CA, USA). Relative quantification of target gene expression was performed using the 2^-ΔΔCt^ comparative method, in which the threshold cycle (CT) value is defined by the point at which there is a statistically significant detectable increase in fluorescence.

### Statistical analysis

Descriptive statistics are presented as the means ± standard deviations or the medians and percentiles (P25-P75). A chi-square test, Fisher exact test, ANOVA, and Kruskal-Wallis test were used to compare groups, as appropriate. Messenger RNA was log transformed to reduce asymmetry. Spearman’s correlation coefficient was used to assess the correlations of podocyte molecules with albuminuria, renal function, and glycemic control. The performance of urinary levels of podocyte-proteins mRNA of patients with diabetes to identify increased albuminuria (≥30 mg/g creatinine) was assessed by the receiver operating characteristic (ROC) curve. The sensitivity, specificity, positive predictive value, and negative predictive value of each gene was calculated. Possion regression analysis was used to test the independent effect of mRNA urinary levels of podocyte-specific proteins (corrected by the glomerular filtration rate) on the development of 'pathological’ albuminuria (dependent variable) in patients with diabetes and prediabetes. These effects were adjusted for confounding factors, such as age, gender, and presence of diabetes. All analyses were performed using SPSS for Windows (version 17.0, SPSS Inc., Chicago, IL), and the level of significance was set at p < 0.05.

## Results

### Demographic and clinical characteristics

Table 1 shows the demographic and clinical characteristics of subjects with prediabetes, diabetes, and the controls. Twenty-one (31%) patients had type 1 DM, while 46 (69%) had type 2 DM. Overall, mean duration of disease at diagnosis was 17 ± 8 years, and for type 1 and type 2 each were 18 ± 7 and 15 ± 8 years, respectively. Overall mean age at diagnosis of DM was 37 ± 15 years, and was 21 ± 11 and 44 ± 19 years for type 1 and type 2, respectively. The prevalence of complications of diabetes was: retinopathy, 48% (15% proliferative) and neuropathy, 36%. The prevalence of NO, MI, and MA in subjects with diabetes was 51%, 21%, and 28%, respectively, giving a prevalence of nephropathy of 49%, as defined by an albumin to creatinine ratio above 30 mg/g.

**Table 1:** Demographic and clinical data of controls, subjects with prediabetes or diabetes

	**Controls (C)**	**Prediabetes (PD)**	**Diabetes (D)**	**p-value**
	**(n = 15)**	**(n = 19)**	**(n = 67)**	
**Age (years)**	40 ± 11	61 ±12	53 ± 13	<0.001
**Gender (female)**	8 (53%)	15 (79%)	42 (63%)	0.267
**Ethnicity (white)**	86.7%	94.7%	73.1%	0.091
**BMI (kg/m**^**2**^**)**	25.1 ± 2.4	31.6 ± 7.7	28.9 ± 6.5	0.016
**SBP (mm Hg)**	116 ± 9	128 ±13	137 ± 18	<0.001
**DBP (mm Hg)**	72 ± 7	77 ±10	80 ± 8	0.006
**Serum creatinine (mg/dL)**	0.86 ± 0.20	0.77 ± 0.10	1.55 ± 1.19	0.011
**eGFR (mL/min/1.73 m**^**2**^**)**	98.8 ± 23.1	95.7 ± 21.8	84.9 ± 39.0	0.038
**Blood glucose (mg/dL)**	90 ± 11	105 ± 11	162 ± 60	<0.001
**Hb1Ac (%)**	5.1 ± 0.6	5.8 ± 0.5	8.7 ± 1.9	<0.001
**Hb1Ac (mmol/mol)**	33 ± 0.6	40 ± 0.5	72 ± 1.9	<0.001
**Albuminuria (mg/g creatinine)**	3.2 (1.5– 6.6)	9.6 (3.7– 6.6)	375.0 (12.0–1340.5)	<0.001

BMI: body mass index; SBP: systolic blood pressure; DBP: diastolic blood pressure; eGFR: estimated glomerular filtration rate; Hb1Ac (glycated hemoglobin): NGSP (%); IFCC (mmol/mol).

Among the subjects with NO, MI, and MA, 54%, 86%, and 78% (p = 0.032), respectively, were on ACE inhibitors or ARB-2. Among the subjects with prediabetes, 56% were on ACE inhibitors or ARB-2. Current treatment of DM consisted of diet therapy, life style modification, and drug therapy: insulin (60%) and/or metformin (50%) and/or glybenclamid (50%).

The mean ages were similar in the prediabetes and diabetes subjects, but the controls were younger. BMI was higher among subjects with prediabetes compared with the controls, and systolic blood pressure (SBP) and diastolic blood pressure (DBP) were also higher in the prediabetes and diabetes subjects. No prediabetes subject had increased albuminuria. Diabetes subjects presented with a lower eGFR compared with the other groups.

### Messenger RNA levels of podocyte-associated proteins in the urine of control, prediabetes, and diabetes subjects

**Figure 1:**
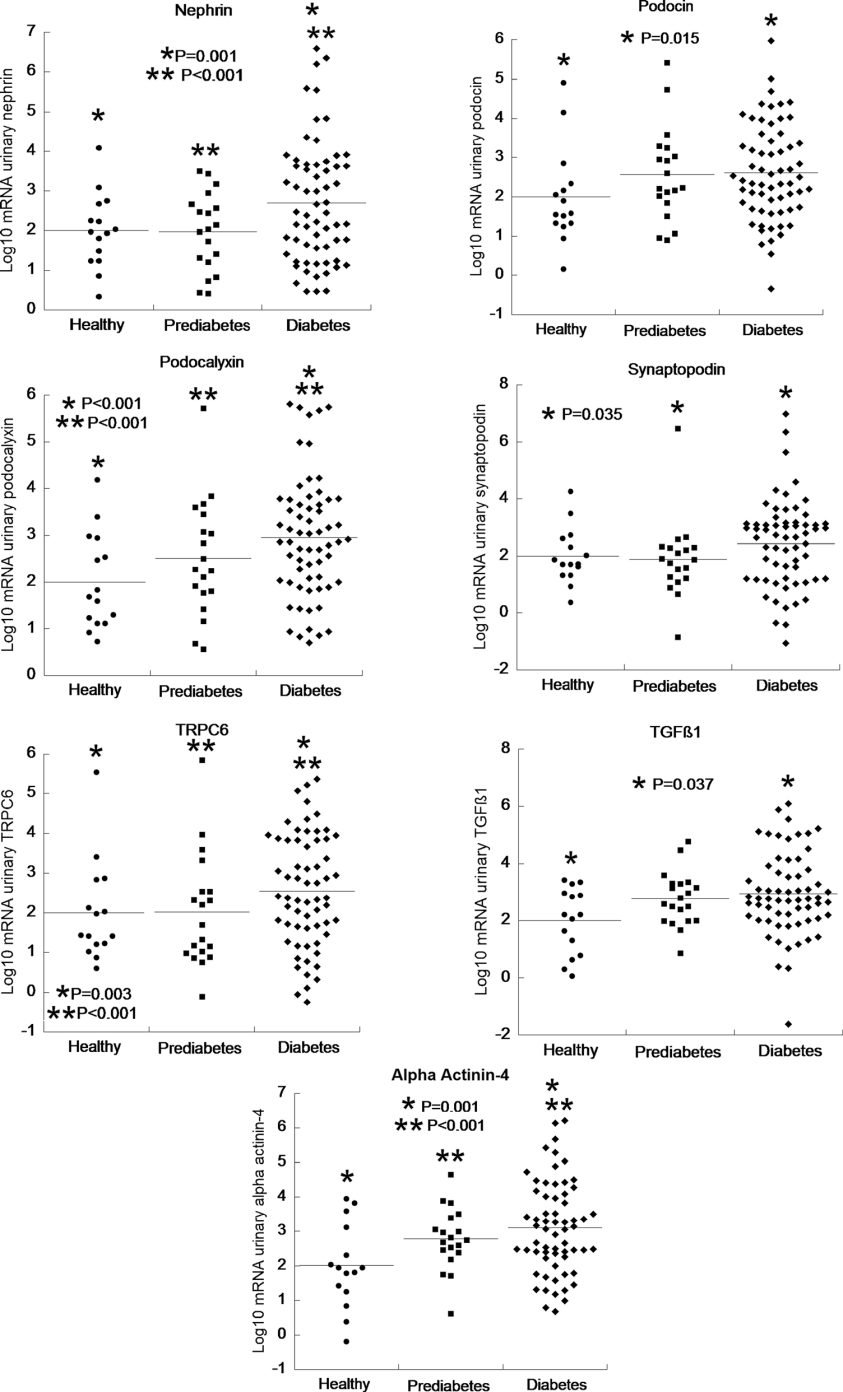
**Dot-plots showing the distribution and means of the log-transformed urinary mRNA of podocyte-associated proteins comparing controls and subjects with prediabetes or diabetes.** Same asterisks (* or **) indicate the comparison between two groups (see P values in the panels).

The urinary levels of the mRNA of all of the genes studied were significantly higher in the diabetes subjects compared with the prediabetes and control subjects (Figure 1). There were no differences in mRNA expression of podocyte markers when comparing patients with type 1 or type 2 DM (data not shown).

Table 2 shows the log-transformed mRNA according to the level of albuminuria (NO, MI, or MA) in the diabetes subjects compared with the prediabetes and control subjects. The expression of nephrin mRNA (Table 2) was significantly increased in the diabetes subjects with NO, MI, or MA compared with the controls and prediabetes subjects. Podocalyxin mRNA in the diabetes subjects at every level of albuminuria was increased compared with the controls; the same was observed in patients with NO and MI compared with subjects with prediabetes. TRPC6 mRNA was higher in patients with MA compared with the subjects with prediabetes and controls and higher in subjects with NO compared with controls. The urinary mRNA of alpha-actinin-4 was higher in patients with NO or MI compared with the subjects with prediabetes and controls. The mRNA expression of podocin, synaptopodin, and TGF-β_1_ did not differ between the five groups.

**Table 2:** Medians and 25th-75th percentiles (P25-P75) of log10 mRNA of podocyte-associated proteins in urine by stage of diabetic nephropathy, in subjects with prediabetes and in controls

				**Diabetes**		
**Log 10 mRNA Urinary**	**Controls (n = 15)**	**Prediabetes (n = 19)**	**Normoalbuminuria (n = 34)**	**Microalbuminuria (n = 14)**	**Macroalbuminuria (n = 19)**	**p-value**
Nephrin	2.03(1.49-2.67)	2.03(1.20-2.93)	2.85(2.14-3.63)	3.35(2.14-5.48)	4.21(3.16-4.95)	<0.001
Podocin	1.57(1.32-2.33)	2.22(1.83-3.25)	2.67(2.12-4.00)	2.54(1.72-3.99)	2.33(1.64-3.62)	0.150
Podocalyxin	1.68(1.18-2.53)	2.10(1.41-2.83)	3.04(2.35-3.76)	3.51(2.79-5.13)	2.89(2.69-3.42)	<0.001
Synaptopodin	1.72(1.30-2.60)	1.86(1.22-2.29)	2.93(1.88-3.12)	2.61(1.13-3.33)	1.62(1.02-4.30)	0.112
TRPC6	1.97(1.21-2.83)	1.69(0.97-2.53)	3.06(1.96-3.83)	2.40(1.89-4.17)	3.06(2.25-4.29)	0.001
Alpha actinin-4	1.94(1.24-3.11)	2.44(1.74-2.67)	3.26(2.48-4.23)	3.54(2.45-4.75)	2.70(2.38-4.08)	<0.001
TGFβ1	2.21(0.78-2.96)	2.59(1.98-3.30)	2.75(2.25-3.86)	2.31(1.40-4.18)	3.02(1.97-4.15)	0.241

Controls: C; Prediabetes: PD; Diabetes with normoalbuminuria: NO; Diabetes with microalbuminuria: MI; Diabetes with macroalbuminuria: MA.

Nephrin: MA vs. C and PD (P<0.001); MI vs. PD and MA vs. NO (P<0.05); MI vs. C, NO vs. C and PD (P<0.05).

Podocalyxin: MI vs. C (P<0.001); MA vs. C, NO vs. C, and MI vs. PD (P<0.05); NO vs. PD (P=0.035).

TRPC6: MA vs. PD and NO vs. PD (P<0.05); MA vs. C (P=0.049).

Alpha actinin-4: NO vs. PD and C (P<0.05); MI vs. PD (P=0.032).

The mRNA of podocin, podocalyxin, and alpha-actinin-4 was higher in the subjects with prediabetes compared with healthy individuals, but these differences were not significant (Table 2). This result is likely due to the small number of patients in each group. Nephrin, synaptopodin, and TGF-β_1_ were similarly expressed in the two groups.

### Correlations between the mRNA levels of podocyte-associated proteins, albuminuria, and glycemic control

Urinary nephrin (*r* = 0.628, p < 0.001), podocalyxin (*r* = 0.452, p < 0.001), and TRPC6 (*r =* 0.455, p < 0.001) were significantly correlated with albuminuria. There was a weak but significant correlation of albuminuria with podocin (*r* = 0.254, p = 0.01) and alpha actinin-4 (*r =* 0.209, p = 0.036). Urinary mRNA expression was also significantly correlated with HbA1C (nephrin: *r =* 0.346, p < 0.001; podocin: *r =* 0.280, p = 0.005; podocalyxin: *r =* 0.441, p < 0.001; TRPC6: *r =* 0.318, p = 0.001; alpha actinin-4: *r =* 0.301, p = 0.002). No correlation was found between either synaptopodin or TGF-β_1_ with albuminuria and glycemic control.

### Effect of clinical variables and mRNA levels on albuminuria

The effect of age, gender, DM, renal function, and mRNA levels of the podocyte-specific proteins on the outcome of albuminuria higher than 30 mg/g creatinine was analyzed. The presence of diabetes, urinary nephrin, TRPC6, and podocin significantly increased the risk of albuminuria in the univariate analysis. However, only diabetes and nephrin remained independent predictors of pathological albuminuria after a multivariate analysis adjusting for age, gender, diabetes, and level of renal function. For every one-unit increase in the log10 mRNA of nephrin, the risk of albuminuria increased by 20% (Table 3).

**Table 3:** Poisson regression analysis of the independent effect of mRNA urinary levels of podocyte-specific proteins on the outcome (albuminuria ≥30 mg/g creatinine) adjusted for age, gender, renal function and presence of diabetes

	**RP**	**95% CI**	**p-value**
**Diabetes**	15.496	2.128–112.867	0.007
**Nephrin**	1.206	1.086–1.338	<0.001
**Podocin**	1.073	0.965–1.193	0.190
**TRPC6**	0.938	0.795–1.108	0.452

Renal function: estimated glomerular filtration rate by the CKD-EPI equation.

### ROC curves for the diagnosis of increased albuminuria

**Figure 2:**
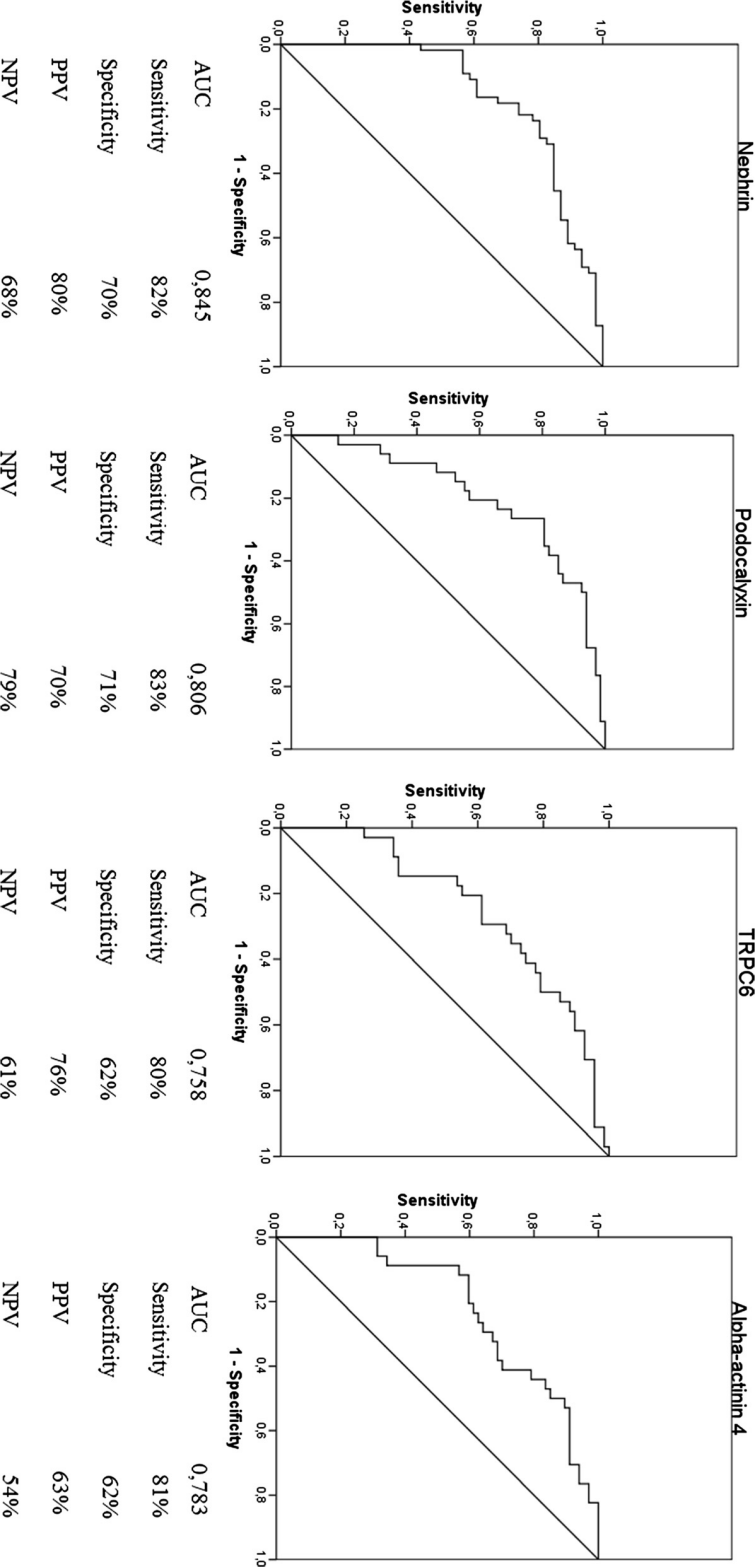
**Diagnostic performance of nephrin, podocalyxin, TRPC6, and alpha-actinin-4 in the diagnosis of albuminuria >30 mg/g creatinine, as determined by the receiver operating characteristic (ROC) curve with related accuracy parameters, expressed as percentages.** AUC: area under the curve; PPV: positive predictive value; NPV: negative predictive value.

The diagnostic performance and accuracy parameters of podocyte-associated proteins for increased albuminuria were determined using the ROC curve shown in Figure 2. Greater areas under the curve and better accuracy were found for nephrin, podocalyxin, TRPC6, and alpha actinin-4 genes than for the other genes. Overall, the sensitivity was greater than 80%, and the specificity ranged between 60% and 70%. Positive predictive values ranged between 63% and 80%, and negative predictive values ranged between 54% and 79%.

Cutoff points to define high or low expressions of gene log10 mRNA in the urine were determined using the ROC curve (Figure 2). The highest sensitivity and specificity were found at 2.61 (nephrin), 2.41 (podocalyxin), 2.14 (TRPC6), and 2.40 (alpha actinin-4). Nephrin discriminated between the increasing levels of albuminuria in diabetes subjects. A high urinary nephrin expression was found in 53%, 71%, and 90% of patients with NO, MI, and MA (p = 0.023), respectively. The proportion of high mRNA expression in each grade of albuminuria was examined for the other podocyte markers, but these differences were not significant.

## Discussion

The pathogenesis of DN is complex and involves the interaction of many hemodynamic and metabolic signaling pathways, such as hyperglycemia, stretch, growth factors, the renin-angiotensin system, and oxidative stress. Functional and structural abnormalities of the glomerular filtration barrier result from these interactions, including the loss of nephrin and α_3_β_1_ integrin, cytoskeletal rearrangement, podocyte detachment, widening of the foot processes, and the accumulation of the mesangial and GBM matrices [2,3,22]. Reductions in the glomerular podocyte number and density have been observed during the early stages of DN, in which podocytopenia predicts the progression of albuminuria and correlates with poor DM control [4,23-25]. Podocyte detachment from the GBM results in podocytopenia and podocyte excretion in the urine as viable or apoptotic cells; this has been demonstrated in DN [14,24] and non-diabetic glomerulopathies [26,27]. Denuded areas in the GBM form synechiae with Bowman’s capsule, which is likely the initial step towards developing progressive glomerulosclerosis and suffering declining renal function [3,6,10].

The increased mRNA levels of podocyte-associated molecules in urine cells can constitute direct evidence of the presence of detached podocytes or their fragments as a result of glomerular injury. Altough anti-podocalyxin antibodies have been used to phenotype podocytes in urine, such method is time-consuming and requires immunocytologic analysis [8,14]. Furthermore, the mRNA of nephrin, podocalyxin, alpha actinin-4, and other podocyte-specific molecules in urine is correlated with podocyte injury and albuminuria and has been shown to be a valuable, non-invasive tool for assessing disease activity and DN progression, primarily in type 2 DM [14,15,28].

There is a lack of information on how podocyte injuries differ in type 1 compared with type 2 DN. Electron microscopy has revealed similar ultrastructural abnormalities in both types of DN. Previous studies have described widened and effaced foot processes, narrowed filtration slit diaphragms, and low podocyte counts and densities, which have been correlated with albuminuria [4,29-31]. Interestingly, these morphologic alterations varied according to the stage of DN: microalbuminuric and proteinuric type 2 diabetes subjects had decreased filtration slit length density per glomerulus and increased foot process width compared with normoalbuminuric patients [29]. We compared the urinary mRNA expression of various podocyte markers between patients with type 1 and type 2 DN, but those levels were essentially the same.

Clinical studies on patients with DN have shown a reduced intra-renal and increased urinary expression of nephrin and alpha-actinin-4, reflecting the loss of podocyte slit diaphragms and cytoskeletal integrity [15,28,32,33]. In addition to nephrin, other proteins can also be suppressed in the renal tissue and/or be increased in the urine of diabetes subjects, including podocin, podocalyxin, synaptopodin [14,15,26,28], and Wilms’ tumor protein (WT-1), a phenotypic marker of mature podocytes [15]. Luminal membrane TRPC6 is also suppressed in experimental DN, as demonstrated by Graham et al. [34], possibly due to the effects of hyperglycemia, reactive oxygen species, and protein kinase C on podocytes. However, the extent to which podocyte proteins or their fragments are lost in the urine and the identities of any specific interchangeable regulatory factors are not yet known.

We found significantly higher mRNA of podocyte-specific molecules in the urine of subjects with diabetes compared with the other groups, which is compatible with podocyturia. However, podocyte excretion did not follow a homogeneous pattern. Interestingly, normoalbuminuric subjects with diabetes showed high mRNA levels of nephrin, TRPC6, and alpha actinin-4 relative to controls, suggesting that podocyte damage may occur early in DN, as has been reported by others [28,33,35]. Patäri et al. [35] proposed that before being shed, the injured podocyte suffers destabilization, altering the podocyte metabolism and leading to the secretion of its molecular components, which can be detected earlier than albuminuria. In agreement with these data, Jim et al. [33] detected nephrinuria using an enzyme-linked immunosorbent assay in 54% of type 2 diabetes subjects with NO, suggesting that urinary nephrin could be a potential pre-clinical biomarker of DN. However, this result still seems controversial because of the damage to the filtration barrier and altered permselectivity may not be an uniform phenomena. For example, Lemley et al. [36] showed that only macroalbuminuric patients with DM type 2 had increased filtrations of high-molecular-weight dextrans through enlarged pores acting as molecular shunts. In patients with lower levels of albuminuria, the shunt size did not differ from that of normal controls.

The cytoskeleton protein alpha-actinin-4 had a pronounced excretion in diabetes subjects with NO and MI that was correlated with poor glycemic control [15,28]. Wang et al. [15] also described an increased expression of alpha-actinin-4 in the urine of patients with DN, while Kimura et al. [37] found a direct correlation between the suppression of alpha-actinin-4 transcripts and mesangial expansion in the biopsies of patients with DN. The disruption of the cytoskeletal dynamics induced by hyperglycemia and advanced glycosylated end-products can cause cytoplasmic translocation and reduced expression of alpha-actinin-4 at the transcriptional level, as demonstrated experimentally in cultured rat podocytes [38]. Podocyte effacement and detachment from the GBM could then follow these broken interactions between the cytoskeleton and the slit diaphragm.

The TRPC6 cation channel causes familial focal segmental glomerulosclerosis in an autosomal-dominant inheritance. This protein may amplify the kidney injury of angiotensin II, causing glomerular cell apoptosis and proteinuria [39]. Thus far, no clinical studies have measured TRPC6 gene expression in the urine of diabetes subjects with incipient or overt nephropathy. We showed that the urinary mRNA of TRPC6 was higher in diabetes subjects than in controls and was positively correlated with albuminuria and HbA1c. It is speculated that the disruption of the TRPC6 molecule and the triggering of Ca^2+^ signaling cascades by mechanical stimuli could lead to loss of integrity of the filtration barrier, owing to altered cytoskeletal dynamics and integrin function, which results in the detachment of podocytes from the GBM [38]. As a result, the increased urinary mRNA of TRPC6 may represent another clinical biomarker of podocyturia in DN. Longitudinal studies are needed to assess the accuracy of TRPC6 during the pre-clinical stages of DN to predict the progression of nephropathy to clinical proteinuria and the progressive loss of renal function.

Overall, podocyturia was correlated with albuminuria and HbA1c, a finding that likely reflects the injury of uncontrolled hyperglycemia and associated mechanisms on the filtration barrier. However, no correlation was observed between podocyturia and eGFR in this cross-sectional design, in contrast with other reports [15,33]. This discrepancy may have occurred because we included subjects with diabetes but with less compromised renal functions (mean eGFR = 85 mL/min/1.73 m^2^) compared with the study of Jim et al. (mean eGFR = 13.3 mL/min/1.73 m^2^) [33] or Wang et al. (53.2 mL/min/1.73 m^2^) [15]. In these two studies, the patients were at more advanced stages of DN, during which foot processes width, podocyte effacement, and GBM thickness were more pronounced, and a correlation with worse renal function was expected.

We found that TGF-β_1_ mRNA expression was augmented in diabetes subjects with MA compared with those with lower levels or normal albuminuria. Pro-fibrotic growth factors play a role in the podocyte damage in DN, as the suppression of TGF-β_1_ has pro-differentiation effects and its induction activates pro-apoptotic signaling pathways and podocyte apoptosis [40]. Wahab et al. [41] reported that the glomerular podocyte expression of TGF-β_1_ increased with DN stage; i.e., TGF-β_1_ expression was lower at an early stage of DN and more pronounced in overt nephropathy, suggesting that TGF-β_1_ induces pro-fibrotic responses and leads to glomerulosclerosis in the long term.

It is not known whether podocyturia represents a very early marker of glomerular epithelial cell injury in subjects with impaired glucose tolerance but not diabetes. Our results showed that urinary mRNA of some podocyte-associated molecules, such as podocin, podocalyxin, alpha actinin-4 and TGF-β_1_, were highly expressed in subjects with prediabetes in relation to the healthy individuals, which could suggest early podocyte damage potentially associated with the metabolic abnormalities these patients present. Differences in mRNA levels, however, did not reach statistical significance, but this lack of association could be related to our small sample size, precluding a more definitive interpretation of our findings. If glucose intolerance per se in the absence of augmented albuminuria does induce early morphologic and functional changes in podocytes, this must be confirmed in another study with a greater number of prediabetes subjects.

Some limitations of our study should be considered. First, the study used a cross-sectional design and included a relatively small sample size. Second, molecular analyses were restricted to the urine sediment, because our patients had classical clinical presentations of DN and did not have a kidney biopsy. However, if we had included biopsies for atypical presentations, such as a faster decline of renal function and/or active urinary sediment, these could have led to skewed podocyte-protein expression of potential non-diabetic glomerulopathies. Third, the effect of angiotensin II inhibitors on the expression of podocyte molecules was not controlled for in this study. The mRNA from podocyte proteins could have been reduced by the concomitant use of these drugs, as previously described either in the urine [18] and the renal tissue [42]. The real magnitude of this effect must be quantified in a longitudinal study.

## Conclusions

In summary, the urinary mRNA levels of podocyte-associated proteins were significantly higher in subjects with diabetes than in subjects without diabetes. Urinary nephrin discriminated between the different stages of DN and predicted increases in albuminuria. This preliminary study did not detect any abnormal urinary expression of podocyte-associated proteins in subjects with prediabetes.

## Abbreviations

DN: Diabetic nephropathy; ESRD: End-stage renal disease; DM: Diabetes Mellitus; MI: Microalbuminuria; NO: Normoalbuminuria; MA: Macroalbuminuria; GBM: Glomerular basement membrane; RT-PCR: Real time polymerase chain reaction; ACE: Angiotensin converting enzyme; ARB: Angiotensin-receptor blocker; UAER: Urinary albumin excretion rate; OGTT: Oral glucose tolerance test; PG: Plasma glucose; eGFR: Estimated glomerular filtration rate; CKD-EPI: Chronic Kidney Disease Epidemiology; ROC: Receiver operating characteristic; HbA1c: Glycated hemoglobin; TRPC6: Transient receptor potential calcium channel 6; TGFβ: Transforming grown factor β; SBP: Systolic blood pressure; DBP: Diastolic blood pressure; WT-1: Wilms’ tumor protein-1.

## Competing interests

The authors declare that they have no competing interests.

## Authors’ contributions

*JF* contributed to the acquisition, analysis, and interpretation of the data, drafted the article, and critically revised the text for important intellectual content; *LHC, FG* drafted the article and critically reviewed it for important intellectual content; *PR, GJ, MS,* and *SP* aided in the acquisition of the data; *FV* participated in the conception and design of the study, acquisition of data, analysis and interpretation of data, drafted the article, reviewed it critically for important intellectual content, and provided final approval of the draft. All authors read and approved the final manuscript.

## Authors’ information

Post Graduate Program in Medicine: Medical Sciences, Universidade Federal do Rio Grande do Sul, Porto Alegre, RS, Brazil; Division of Nephrology, Hospital de Clínicas de Porto Alegre, Porto Alegre, RS, Brazil; Division of Endocrinology, Hospital de Clínicas de Porto Alegre, Porto Alegre, RS, Brazil. Adress: Rua Ramiro Barcelos, 2350, Room 2030, second floor, Porto Alegre, RS 90035–003, Brazil.

## Pre-publication history

The pre-publication history for this paper can be accessed here:

http://www.biomedcentral.com/1471-2369/14/214/prepub
